# Attenuated NOX2 expression impairs ROS production during the hypoinflammatory phase of sepsis

**DOI:** 10.1186/cc11738

**Published:** 2012-11-14

**Authors:** L Kuchler, V Morbitzer, A Heeg, LK Eifler, T Knape, B Brüne, A von Knethen

**Affiliations:** 1 Institute of Biochemistry I - Pathobiochemistry, Goethe-University Frankfurt, Germany; 2 Fraunhofer IME Project Group - Translational Medicine and Pharmacology, Frankfurt, Germany

## Background

The multicomponent phagocytic NADPH oxidase produces reactive oxygen species (ROS) after activation by microorganisms or inflammatory mediators [1]. In the hypoinflammatory phase of sepsis, macrophages are alternatively activated by contact with apoptotic cells or their secretion products. This inhibits NADPH oxidase and leads to attenuated ROS production [2] and furthermore contributes among others to a hyporeactive host defense. Due to this immune paralysis, sepsis patients suffer from recurrent and secondary infections [3]. We focused on the catalytic subunit of NADPH oxidase, the transmembrane protein NOX2 [4]. We assume that after induction of sepsis the expression of NOX2 is reduced and hence ROS production is decreased.

## Methods

We induced polymicrobial sepsis in mice by cecal ligation and puncture. The ability of peritoneal macrophages (PMs) to produce ROS was determined by FACS via hydroethidine assay. NOX2 expression of PMs was determined by western blot and qPCR. To elucidate the mechanism causing mRNA destabilization, we performed *in vitro *experiments using J774 macrophages. To obtain an alternatively activated phenotype, macrophages were stimulated with conditioned medium from apoptotic T cells (CM). By luciferase assays we figured out a 3'UTR-dependent regulation of NOX2 mRNA stability. Assuming that a protein is involved in the mRNA degradation, we performed a RNA pulldown with biotinylated NOX2-3'UTR constructs followed by mass spectrometry. We verified the role of SYNCRIP by siRNA approach. Additionally, we overexpressed NOX2 in J774 cells and analyzed the ROS production (w/wo CM treatment) by FACS.

## Results

We found an impaired expression of NOX2 at RNA and protein level along with decreased ROS production after induction of sepsis in mice as well as stimulating J774 macrophages with CM of apoptotic T cells. This is due to a time-dependent NOX2 mRNA degradation depending on SYNCRIP, a RNA-binding protein, which stabilizes NOX2 mRNA through binding to its 3'UTR under normal conditions. In line, knockdown of SYNCRIP also decreases NOX2 mRNA expression. We assume that a CM-dependent modification or degradation of SYNCRIP prevents its stabilizing function. As the overexpression of NOX2 restores ROS production of CM-treated J774 cells, we assume that NOX2 expression is crucial for maintaining NADPH activity during the hypoinflammatory phase of sepsis.

## Conclusion

Our data imply a regulatory impact of SYNCRIP on NOX2 stability during the late phase of sepsis. Therefore, further understanding of the regulation of NADPH oxidase could lead to the design of a therapy to reconstitute NADPH oxidase function, finally improving immune function in sepsis patients.
